# Organic cation/carnitine transporter (OCTN2) interaction proteome in rat astrocytes: Role of phosphatase PP2A

**DOI:** 10.1186/2193-1801-4-S1-P30

**Published:** 2015-06-12

**Authors:** Katarzyna Nalecz, Barbara Juraszek

**Affiliations:** Nencki Institute of Experimental Biology, Poland

**Keywords:** astrocytes, membrane transporter, protein phosphatase

L-carnitine is essential for translocation of fatty acids for their mitochondrial oxidation, a process shown in the brain to take place in astrocytes. However, it is not synthesized in the brain and has to be transported to brain cells. Organic cation/carnitine transporter novel family member 2 - OCTN2 (SLC22A5) presence was shown in endothelial cells forming the blood-brain barrier, as well as in neurons and astrocytes. We showed that OCTN2 activity in astrocytes and its presence in plasma membrane are higher upon activation of protein kinase C (PKC), but no phosphorylation of OCTN2 was detected. Therefore, we aimed to identify OCTN2-interacting partners and to define their role in transporter regulation. Mass spectrometry analysis identified several cytoskeletal, ribosomal, mitochondrial, and heat-shock proteins as well as the proteins involved in signaling pathway and trafficking. We focused on protein phosphatase PP2A subunits identified in OCTN2 proteome and we observed co-precipitation of OCTN2 with PP2A structural (A) and catalytic (C) subunits, as well as with two regulatory subunits – striatin and SG2NA. Activation of PKC with phorbol ester (PMA) did not change the amount of co-precipitating subunits A and C but significantly lowered the amount of co-precipitating SG2NA. Immunocytochemistry analysis of astrocytes showed OCTN2 co-localization with PP2A C subunit and with SG2NA in vesicular structures in the cytoplasm. PMA treatment did not change this co-localization, although an augmented amount of OCTN2 was detected in plasma membrane. We postulate that interaction of OCTN2 with PP2A arrests the transporter in cytoplasm in dephosphorylated state, while PKC activation releases SG2NA subunit from the complex, resulting in transporter trafficking to the cell surface.

